# Supplementary material for: Genetic tracking of Crimean-Congo haemorrhagic orthonairovirus in Hyalomma population infesting cattle in Nigeria
Source: PLoS One. 2025 Jan 24;20(1):e0316770. doi: 10.1371/journal.pone.0316770 (PMC11761185; doi:10.1371/journal.pone.0316770)
Supplement: Attachment — Submitted filename: Response to Reviewers comments_Hyalomma ticks_03102024.docx [file pone.0316770.s001.docx]

Reviewer #1: Comments on the Manuscript:

The manuscript contains original data on the strain of the CCHF virus in Nigeria. However, I would like to draw attention to some methodological issues:

1. Sample Collection:
- The samples were collected from cattle brought to the slaughterhouse. However, there is no information about the location from which these cattle were brought.
- It is unclear whether all the cattle were grazing on pastures or if they were sourced from intensive farming operations. This information is crucial for understanding the epidemiological context.
**RESPONSE:** Locations and information about sources of cattle and their rearing system are now inserted in the manuscript (Lines 106-109)

2. Sampling Period:
- The authors collected samples between December and March. The rationale behind choosing this particular timeframe has not been provided. Why were samples collected specifically during these months? Are these the months when Hyalomma ticks are active or host seeking session? If so, please provide reference.
**RESPONSE:** This is the observed active months for Hyalomma tick, hence this period was selected. This statement has been made and equally referenced. (Lines 116-119)

3. Tick Collection Data:
- The manuscript should include the minimum and maximum number of ticks collected from each animal.
- Additionally, it would be beneficial to provide a table detailing the number of ticks collected from each animal by month, including the species of ticks identified.
**RESPONSE:** The minimum and maximum numbers of ticks obtained per animal is now indicated (Lines 136). However, creating a table showing number of ticks collected for each of the 318 cattle will make the manuscript boring. We belief that Tables 1 and 2 have given a good summary of the Hyalomma species and developmental stages. It further provided information on the variables of infested cattle.

4. cDNA Synthesis Verification:
- The entire tick pools could have been checked for housekeeping genes post-cDNA synthesis. How did the authors ensure that the cDNA synthesis was accurate?
**RESPONSE:** The cDNA synthesis was conducted on both test samples and positive control. For this conventional amplification, housekeeping genes were not tested.

5. Specific Comments on Table 2:
- Please provide more information regarding the SG abbreveation in Table 2.
**RESPONSE:** Full meaning of the abbreviation is now inserted. (Line 234)

6. Serological Status of Cattle:
- Why were the cattle not found to be seropositive? How do the authors explain this finding?
**RESPONSE:** Possible reasons for the non-detection of the targeted region of CCHFV S-gene segment has already been indicated (Lines 342-346). Note that this study does not include antibody detection.

7. Minor Corrections:
- Line 339: "hyalomma" should be corrected to "Hyalomma".
**RESPONSE:** Corrected. Thanks (Line 338)

Reviewer #2: CCHFV is a zoonotic pathogen known to cause severe infections in humans in close contact with animals that serve as amplifying hosts and are infested with tick vectors. The study presents interesting data that adds knowledge to the distribution of the virus in Nigeria.
There are some minor changes that need to be made by the authors.

Here are some comments to consider:

Abstract:
L35: The percentages of the Hyalomma species add up to 100.4%, not 100%. Kindly check and adjust.
**RESPONSE:** This error has been corrected. Thanks so much for this keen observation. (Line 35)

Introduction:
L77: Change “leaved” to “lived”.
L89: Change “hyalomma” to “Hyalomma”.
**RESPONSE:** These typos have been corrected (Lines 77 & 89)

Tick collection and processing:
L133-L140: I assume all ticks present on the cattle were collected, and sent to the laboratory for identification and only the Hyalomma species processed for RNA extraction. If so, I suggest the section reflects exactly that.
**RESPONSE:** Only Hyalomma ticks were obtained from the cattle in this study. We did this as Hyalommas, being the principal CCHFV vector, are likely to indicate the current status of this virus after more than a decade of failure to know its current status in its vector. Our previous study (unpublished) and other research groups failed to identify CCHFV in tick species infesting cattle (including Hyalomma ticks)

Sequencing and phylogenetic analysis:
L172-L172: I recommend changing the sentence to “The sequence generated was subsequently assembled using DNASTAR Lasergene v. 7 and a BLAST performed in NCBI
to identify related strain”.
**RESPONSE:** Corrected as suggested. Thanks for identifying the typo. (Lines 174-175)

Results:
If all tick species were collected, please indicate the different species identified and narrow down to the Hyalomma species. Again, why were other tick species such as Amblyomma variegatum not screened for CCHFV? These have been reported to carry the virus and could potentially contribute to its circulation in Nigeria.

**RESPONSE:** In addition to the previous response, this study was conducted to guide future studies to be conducted within and countries sharing international borders with Nigeria. Previous studies in Nigeria had been met with failure to identify CCHFV in all tick species (including *A. variegatum*). Hence, focusing on the known principal vector was our first approach to answer this decade-long query of CCHFV strains circulating in tick vector.

L187: Again, the percentages of the Hyalomma species add up to 100.4%, not 100%. Kindly check and adjust.
**RESPONSE:** Thanks. This has been recalculated as previously indicated (Line 191)

Table 2: Kindly ensure that all the Breed is captured in the key.
**RESPONSE:** All the Breed are now captured in the key i.e., SG- Sokoto Gudali (Lines 234)

L253: Please revise the sentence to be clear and easy to read.
**RESPONSE:** This has been reframed. (Line 251)

Discussion:
L334–335: The percentages of the Hyalomma species add up to 100.4%, not 100%. Kindly check and adjust.

**RESPONSE:** This has been corrected as shown in Lines 333 -334

L337: Change “hyalomma” to “Hyalomma”.
L339: Change “hyalomma” to “Hyalomma”.

**RESPONSE:** This has been corrected as shown in Lines 336 & 338

L358-359: The sentence should be revised to; “It should be noted that this study was limited, in that individual ticks that made up each pool were not tested (to know the actual number of positive ticks).

**RESPONSE:** The comma has been inserted. Thanks (Line 357)

L381: Change “characterised” to “characterise”
**RESPONSE:** The changes have been made. (Line 380)

I suggest you include some limitations of the study at the end of the discussion. By way of recommendation, you can suggest that studies sequence the whole genome of CCHFV to determine if there is reassortment in any of the segments. Reassortment in the M segment for instance, has been reported to influence the pathogenicity and epidemiology of the virus.
Reference: Burt, F. J., Paweska, J. T., Ashkettle, B., & Swanepoel, R. (2009). Genetic relationship in southern African Crimean-Congo haemorrhagic fever virus isolates: Evidence for occurrence of reassortment. Epidemiology and Infection, 137(9), 1302–1308. <https://doi.org/10.1017/S0950268808001878>

**RESPONSE:** This has been entrenched in the penultimate statement in the discussion section. (Lines 380-381)
